# Clinicopathological Correlation and Prognostic Significance of Protein Kinase Cα Overexpression in Human Gastric Carcinoma

**DOI:** 10.1371/journal.pone.0056675

**Published:** 2013-02-26

**Authors:** Shee-Chan Lin, Wei-Yu Chen, Kai-Yuan Lin, Sheng-Hsuan Chen, Chun-Chao Chang, Sey-En Lin, Chia-Lang Fang

**Affiliations:** 1 Division of Gastroenterology, Department of Internal Medicine, Mackay Memorial Hospital, Taipei, Taiwan; 2 Department of Pathology, School of Medicine, College of Medicine, Taipei Medical University, Taipei, Taiwan; 3 Department of Pathology, Wan Fang Hospital, Taipei Medical University, Taipei, Taiwan; 4 Department of Medical Research, Chi-Mei Medical Center, Tainan, Taiwan; 5 Department of Biotechnology, Chia Nan University of Pharmacy and Science, Tainan, Taiwan; 6 Department of Internal Medicine, School of Medicine, College of Medicine, Taipei Medical University, Taipei, Taiwan; 7 Division of Gastroenterology, Department of Internal Medicine, Taipei Medical University Hospital, Taipei, Taiwan; 8 Department of Pathology, Taipei Medical University Hospital, Taipei, Taiwan; National Cancer Center, Japan

## Abstract

**Objectives:**

This study investigated the PKCα protein expression in gastric carcinoma, and correlated it with clinicopathological parameters. The prognostic significance of PKCα protein expression in gastric carcinoma was analyzed.

**Methods:**

Quantitative real-time PCR test was applied to compare the PKCα mRNA expression in tumorous and nontumorous tissues of gastric carcinoma in ten randomly selected cases. Then PKCα protein expression was evaluated in 215 cases of gastric carcinoma using immunohistochemical method. The immunoreactivity was scored semiquantitatively as: 0 = absent; 1 = weak; 2 = moderate; and 3 = strong. All cases were further classified into two groups, namely PKCα overexpression group with score 2 or 3, and non-overexpression group with score 0 or 1. The PKCα protein expression was correlated with clinicopathological parameters. Survival analysis was performed to determine the prognostic significance of PKCα protein expression in patients with gastric carcinoma.

**Results:**

PKCα mRNA expression was upregulated in all ten cases of gastric carcinoma via quantitative real-time PCR test. In immunohistochemical study, eighty-eight out of 215 cases (41%) of gastric carcinoma revealed PKCα protein overexpression, which was statistically correlated with age (*P* = 0.0073), histologic type (*P*<0.0001), tumor differentiation (*P* = 0.0110), depth of invasion (*P* = 0.0003), angiolymphatic invasion (*P* = 0.0373), pathologic stage (*P* = 0.0047), and distant metastasis (*P* = 0.0048). We found no significant difference in overall and disease free survival rates between PKCα overexpression and non-overexpression groups (*P* = 0.0680 and 0.0587). However, PKCα protein overexpression emerged as a significant independent prognostic factor in multivariate Cox regression analysis (hazard ratio 0.632, *P* = 0.0415).

**Conclusions:**

PKCα protein is upregulated in gastric carcinoma. PKCα protein expression is statistically correlated with age, histologic type, tumor differentiation, depth of invasion, angiolymphatic invasion, pathologic stage, and distant metastasis. The PKCα protein overexpression in patients with gastric carcinoma is a significant independent prognostic factor in multivariate Cox regression analysis.

## Introduction

Gastric cancer is the fourth most common cancer worldwide, and the second leading cause of cancer death in men and the fourth in women [1], [2]. Although surgical techniques and adjuvant chemotherapy have substantially improved recently and rate of early detection by endoscopy has increased, the overall 5-year survival rate remains dismal [1]. A steady decline in gastric cancer incidence has been observed in most developed countries and some developing countries over the past 50 years [2]. However, gastric cancer remains a major public health problem throughout the world. The carcinogenesis of gastric carcinoma is not well understood, but it exhibits a multi-hit process of genetic alterations involving suppressor genes and oncogenes [3], [4].

The protein kinase C (PKC) family consists of serine-threonine kinases that act by phosphorylating their specific protein substrates. The PKC family members are classified into three major groups: classical (α, β, and γ), novel (δ, ε, η, and θ), and atypical (μ, λ, ξ). Activation of classical PKCs depends on calcium and phospholipids. Novel PKCs are activated by phospholipids, and activation of atypical forms occurs independently of calcium or phospholipids. PKCs are involved in various cellular processes including regulating gene expression, proliferation, differentiation, apoptosis, migration, and tumor development [5]–[10]. Because of the existence of many PKC isoforms and their involvement in different cellular signaling pathways, the roles of PKC isoforms in carcinogenesis have not been clarified [8].

Among the PKC isoforms, PKCα is ubiquitously expressed in many tissues and has been associated with cell proliferation, apoptosis, and cell motility. PKCα activation results in increased cell motility and invasiveness in in vivo and in vitro cancer models [8]. PKCα has been found to be the most important PKC isoform in the formation and progression of malignancies in various cell lines [11]. Abnormal levels of PKCα have been found in transformed cell lines and human cancers [12]. Substantial evidence from gene knockout studies indicates that PKCα activity regulates cancer growth and progression. Selective targeting of PKCα thus has a potential therapeutic role in a wide variety of human cancers [13].

The specific role of PKCα in gastrointestinal tumors has not been well studied [14]. Among the PKC family, PKCα is the most abundant isoform in gastric epithelia, and might play an important role in the carcinogenesis and metastasis of gastric cancers [10]. Furthermore, PKCα is known to play a critical role in cancer cell proliferation and in maintaining the transformed phenotype and tumorigenic capacity of gastric cancer cells [10], [14]. Our previous study using quantitative real-time PCR tests demonstrated that in gastric carcinoma, PKCα mRNA overexpression was correlated with distant metastasis, and might be an independent prognostic marker [15]. However, the expression of PKCα protein in gastric carcinoma and its clinicopathological correlations have not been investigated. Our study thus evaluated the expression of PKCα protein in gastric carcinoma using immunohistochemical method. The aims of this study were to assess the expression of PKCα protein in gastric carcinoma, and to correlate it with other clinicopathological parameters. The prognostic significance of PKCα protein overexpression in gastric carcinoma was also investigated.

## Materials and Methods

We collected 215 consecutive cases of gastric carcinoma from the medical files of both Wan-Fang Hospital and Taipei Medical University Hospital in Taiwan. All patients included in our study group were treated between 1997 and 2011, and had received surgical resection with radical total or subtotal gastrectomy and lymph node dissection. All pathological reports and hematoxylin & eosin sections were available and reviewed to determine pathological parameters including tumor size, location, histologic type, differentiation, depth of invasion, angiolymphatic invasion, nodal status, local recurrence status, distant metastasis, and pathologic staging. The pathologic staging was based on the 7th edition of the TNM staging system of AJCC. For each case, one or more representative sections and corresponding blocks of both tumorous and non-tumorous tissues were retrieved for immunohistochemical study. From the patients’ records, we obtained the information including postoperative courses, tumor recurrence, distant metastasis, and outcome. This study received ethical approval from the institutional review board of Taipei Medical University. Written informed consent was obtained from each participant before tissue acquisition.

### Quantitative Real-Time PCR Test

At first quantitative real-time PCR test was applied to test and compare the mRNA expression of PKCα in tumorous and nontumorous tissues of gastric carcinoma in a small scale. Ten tumor and non-tumor pairs of gastric tissues were randomly selected from the Tumor and Serum Bank of Chi-Mei Medical Center (Tainan, Taiwan). All samples were collected from the specimens via radical gastrectomy. The non-tumor part was taken from the grossly normal gastric mucosa away from the tumor. All tissues were frozen in liquid nitrogen within 20 min and kept at –80°C until use.

The procedure of quantitative real-time PCR test was performed according to previous study [15].

### Immunohistochemical Study

Sections of 5 µm thickness were taken from formalin-fixed paraffin-embedded blocks. The procedure of immunohistochemical study was performed according to previous study [15]. Deparaffinized sections were incubated in pH 6.0 citrate buffer for 40 min at 95°C on a hotplate to retrieve the antigens. Endogenous peroxidase was blocked by 3% hydrogen peroxide for 5 min. The sections were subsequently incubated with antibody against PKCα (Santa Cruz Biotechnology Inc., Santa Cruz, CA, SC-8393) for 30 min at room temperature at a dilution of 1∶100 using DAKO primary antibody diluent. To detect immunoreactivity, the avidin-biotin-complex method was applied according to the manufacturer’s instructions. A sensitive Dako EnVision kit (Dako North America Inc., Carpinteria, CA) was used as the detection system. After incubation with secondary antibody (DAKO EnVision) for 30 min at room temperature, followed by diaminobenzidine for 8 min, sections were counterstained with Mayer’s hematoxylin. Normal human distal renal tubules were used as a positive control. The negative control was made by omitting the primary antibody and incubation with PBS.

The PKCα immunoreactivity was evaluated independently by two pathologists (CL Fang and SE Lin). As in previous studies [16], [17], the results were scored semiquantitatively in four categories: 0 = absent, 1 = weak, 2 = moderate, and 3 = strong immunoreactivity. The positive staining of nerve bundles in the same slide was used as the positive internal control and was allocated score 2. The negative control provided a reference of score 0. Score 1 was defined as positive staining that was weak compared with internal control; score 3 was allocated to positive staining stronger than that of internal control. Finally each case was assigned to one of two groups: either PKCα overexpression with score 2 or 3, or non-overexpression with score 0 or 1.

### Statistical Analysis

All data were analyzed using the SAS software (Version 9.2 SAS Institute Inc., Cary, NC). Chi-square tests and correlation coefficient analysis were performed to determine whether the correlations between PKCα overexpression and other clinicopathological parameters were statistically significant. The cumulative overall survival rates and disease free survival rates were calculated by the Kaplan-Meier method, and the differences in survival rates between PKCα overexpression and non-expression groups were analyzed by a log-rank test. To determine the relative prognostic impact of PKCα overexpression compared with other established prognostic markers, overall survival was analyzed using the Cox proportional hazard model. For uni and multivariate Cox regression analysis, continuous variables were coded as binary variables. Backward multivariate analysis was also applied to identify independent prognostic markers. All tests were performed with the significance level at *P*<0.05.

## Results

### PKCα mRNA Expression was Upregulated in Gastric Carcinoma

In all ten tumor and non-tumor pairs of gastric tissues randomly selected for quantitative real-time PCR, the mRNA expression of PKCα in tumor tissues were substantially increased when compared to non-tumor tissues (Table 1).

**Table 1:** Quantification of PKCα mRNA Expression by Quantitative Real-Time PCR in 10 Tumor and Non-tumor Pairs of Gastric Tissues.

	Non-tumor			Tumor		
No.	PKCα	GAPDH	Δ*C_non-tumor_*	PKCα	GAPDH	Δ*C_tumor_*
1	35.10	25.14	9.96	32.09	23.41	8.68
2	34.90	28.17	6.73	28.87	23.89	4.98
3	34.84	30.15	4.69	33.32	30.00	3.32
4	40.00	29.58	10.42	34.53	28.05	6.48
5	34.91	31.22	3.69	33.21	31.18	2.03
6	40.00	23.54	16.46	40.00	24.83	15.17
7	34.44	32.29	2.15	29.11	28.61	0.50
8	40.00	31.02	8.98	33.71	26.64	7.07
9	35.40	28.47	6.93	33.01	27.63	5.38
10	35.73	27.25	8.48	33.32	26.83	6.49

### Basic Data for Immunohistochemical Study

Data from a total of 215 cases of gastric carcinoma were analyzed. The patients included 134 men and 81 women, with a mean age of 69 years (range 30 years to 96 years). Among the 215 cases, 52 patients had the disease at stage I, 43 patients at stage II, 98 patients at stage III, and 22 patients at stage IV. Postoperative clinical follow-up and survival analysis were recorded in all 215 patients. The follow-up period ranged from 5 days to 5131 days (mean 1143 days). Distant metastasis status was obtained in all patients, of whom 67 had metastatic diseases.

### PKCα Protein Expression was Upregulated in Gastric Carcinoma

**Figure 1:**
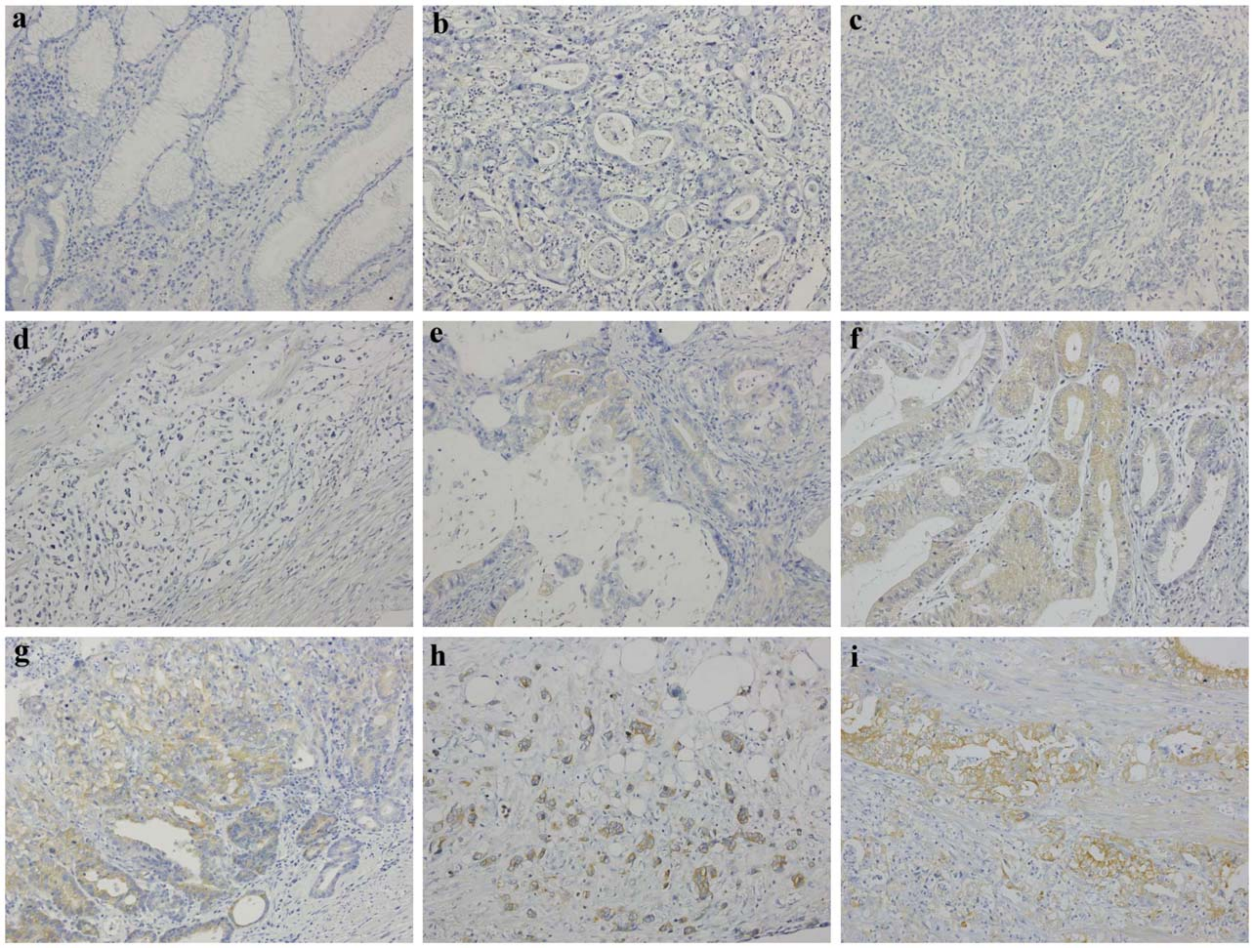
PKCα immunoreactivity in gastric carcinoma of various histologic type and differentiation. **a** normal gastric glands showing negative immunostaining, **b** negative immunostaining in a moderately-differentiated adenocarcinoma of intestinal type, **c** negative immunostaining in a poorly-differentiated adenocarcinoma of intestinal type, **d** negative immunostaining of signet-ring cells in a diffuse type adenocarcinoma, **e** weakly positive immunostaining in a moderately-differentiated adenocarcinoma of intestinal type, **f** moderately positive immunostaining in a well-differentiated adenocarcinoma of intestinal type, **g** moderately positive immunostaining in a moderately to poorly-differentiated adenocarcinoma of intestinal type, **h** moderately positive immunostaining in a diffuse type adenocarcinoma, and **i** strongly positive immunostaining in a moderately-differentiated adenocarcinoma of intestinal type. Magnification: X200.

Of the total 215 cases of gastric carcinoma, 88 patients (41%) revealed PKCα protein overexpression. The intensity and distribution of immunoreactivity varied among the PKCα-positive cases, and immunoreactivity was observed in the cytoplasm of the tumor cells. In all cases, the normal gastric glands in non-tumor tissues revealed negative staining (Fig. 1a). Overexpression of PKCα protein was observed in tumor cells but not in normal gastric glands, with the difference being statistically significant (McNemar test, *P*<0.001).

### Overexpression of PKCα Protein Was Statistically Correlated with Age, Histologic Type, Tumor Differentiation, Depth of Invasion, Angiolymphatic Invasion, Pathologic Stage, and Distant Metastasis

A Chi-square test was performed to determine the significance of the difference between PKCα overexpression and other clinicopathological parameters (Table 2). PKCα protein overexpression was statistically correlated with age. Patients aged 60 years or older had a higher rate of PKCα protein overexpression (46%) than those of less than 60 years (25%). There was a statistically significant correlation between PKCα protein overexpression and histologic type (*P*<0.0001). Among 137 cases of intestinal type carcinoma, 71 cases (52%) showed PKCα protein overexpression. In contrast, only 17 out of 78 cases (22%) of diffuse type carcinoma showed PKCα protein overexpression. In addition, overexpression of PKCα protein was significantly statistically correlated with tumor differentiation (*P* = 0.0110). Among the 112 cases of well to moderately-differentiated carcinoma, 55 (49%) displayed PKCα protein overexpression. However, among the 103 cases of poorly-differentiated carcinoma, only 33 (32%) exhibited PKCα protein overexpression. Our data thus revealed that well to moderately-differentiated intestinal type tumors more frequently expressed PKCα protein than those of the diffuse type. The PKCα immunostaining patterns of various histologic type and tumor differentiation are shown in Figs. 1b to 1i. A statistical significance was also noticed between PKCα protein overexpression and depth of tumor invasion. In 66 cases of T1 and T2 tumor (invasion not beyond muscularis propria), 39 (59%) presented PKCα protein overexpression. In contrast, 49 out of 149 cases (33%) of T3 and T4 tumor (invasion of subserosa or deeper) displayed PKCα protein overexpression. Also found is a statistic significance between PKCα protein overexpression and angiolymphatic invasion. There were 135 cases with angiolymphatic invasion and 80 cases with no invasion. The PKCα protein overexpression rates were 36% in the former and 50% in the latter, respectively. The tumors with vascular emboli had lower PKCα protein overexpression rate than those with no emboli. Overexpression of PKCα protein has a statistical correlation with pathologic stage. Among the 95 stage I and II cases, there were 49 (52%) with PKCα protein overexpression. In 120 cases at stage III and IV, only 39 (33%) revealed PKCα protein overexpression. We observed that early stage tumors were likely to express PKCα protein than tumors with advanced stage. Finally, there was a significantly statistical correlation between PKCα protein overexpression and distant metastasis. Eighteen out of 67 cases (27%) with distant metastasis showed overexpression of PKCα protein, and 70 out of 148 cases (47%) with no distant metastasis possessed PKCα protein overexpression. Therefore, PKCα protein overexpression was negatively statistically correlated with distant metastasis. In addition, correlation coefficients were calculated. The correlation coefficient (r) and P value (*P*) in statistically significant variables were as follows: age (r = 0.16301; *P* = 0.0167), histologic type (r = –0.29364; *P*<0.0001), tumor differentiation (r = –0.17341; *P* = 0.0109), depth of invasion (r = –0.24581; *P* = 0.0003), angiolymphatic invasion (r = –0.14199; *P* = 0.0375), pathologic stage (r = –0.19269; *P* = 0.0046), and distant metastasis (r = –0.19245; *P* = 0.0046).

**Table 2:** PKCα Protein Expression in Gastric Carcinoma and its Correlation with Clinicopathological Parameters.

	PKCα overexpression		
Parameters	Negative	Positive	*P* *
	(case number)	(case number)	
Age (years)			
<60	39	13	
≧60	88	75	0.0073
Gender			
Female	46	35	
Male	81	53	0.5971
Tumor size (cm)			
≤5	55	48	
>5	72	40	0.1048
Tumor location			
Proximal	20	17	
Distal	107	71	0.4953
Histologic type			
Intestinal type	66	71	
Diffuse type	61	17	<0.0001
Differentiation			
Well to moderately	57	55	
Poorly	70	33	0.0110
Depth of invasion			
T1–T2	27	39	
T3–T4	100	49	0.0003
Angiolymphatic invasion			
Absent	40	40	
Present	87	48	0.0373
Nodal status			
N0	38	33	
N1-3	89	55	0.2453
TNM stage			
I+II	46	49	
III+IV	81	39	0.0047
Distant metastasis			
Absent	78	70	
Present	49	18	0.0048
Local recurrence			
No	113	79	
Yes	14	9	0.8526

* Significance level: *P*<0.05.

No statistical significance was found between PKCα protein overexpression and other clinicopathological parameters including gender, tumor size, location, lymph node status, and local recurrence.

### The Expression of PKCα Protein Was a Significant Independent Prognostic Factor in Multivariate Cox Regression Analysis

The data of 215 patients were enrolled for survival analysis. The overall survival rate among the 88 patients with PKCα protein overexpression was 64%, and among the 127 without overexpression was 47%. We also analyzed disease free survival. In PKCα protein overexpression group and non-overexpression group, the disease free survival rates were 58% and 42%, respectively. The difference in overall and disease free survival rates between the PKCα overexpression and non-overexpression groups was not statistically significant (log rank test *P* = 0.0680 and 0.0587), but did indicate a tendency for patients with PKCα protein overexpression to have a longer overall survival and disease free survival than those lacking overexpression.

The univariate Cox regression analysis of prognostic markers is summarized in Table 3. The overall survival was statistically correlated with age, tumor size, histologic type, tumor differentiation, depth of invasion, angiolymphatic invasion, nodal status, pathologic staging, local recurrence, and distant metastasis. PKCα protein overexpression was not statistically correlated with overall survival in univariate analysis (*P* = 0.0699). However, backward multivariate Cox regression analysis found that PKCα protein overexpression was an independent prognostic marker for overall survival. Patients in the overexpression group had a statistically significant longer overall survival rate compared with patients in the non-expression group (hazard ratio 0.632; 95% confidence interval 0.407–0.982; *P* = 0.0415) (Table 4). Other co-variables of prognosis included age, pathologic stage, local recurrence, and distant metastasis.

**Table 3:** Uni-Variate Analysis of Prognostic Markers in 215 Patients with Gastric Carcinoma.

Variables	Hazard ratio	95% CI*	*P* **
Age (years)			
<60	1		
≧60	1.914	1.158–3.164	0.0114
Gender			
Female	1		
Male	1.267	0.837–1.920	0.2632
Tumor size (cm)			
≤5	1		
>5	3.396	2.163–5.333	<0.0001
Tumor location			
Proximal	1		
Distal	0.682	0.417–1.114	0.1264
Histologic type			
Intestinal type	1		
Diffuse type	1.525	1.023–2.274	0.0381
Differentiation			
Well to moderately	1		
Poorly	1.761	1.183–2.620	0.0053
Depth of invasion			
T1–T2	1		
T3–T4	6.497	3.262–12.940	<0.0001
Angiolymphatic invasion			
Absent	1		
Present	3.813	2.297–6.328	<0.0001
Nodal status			
N0	1		
N1-3	6.281	3.343–11.800	<0.0001
Pathologic stage			
I+II	1		
III+IV	6.147	3.627–10.420	<0.0001
Distant metastasis			
Absent	1		
Present	5.224	3.435–7.944	<0.0001
Local recurrence			
No	1		
Yes	3.494	2.117–5.766	<0.0001
PKCα overexpression			
Negative	1		
Positive	0.677	0.444–1.032	0.0699

* CI: confidence interval;

** Significance level: *P*<0.05.

**Table 4:** Backward Multi-Variate Analysis of PKCα Protein Expression and Other Prognostic Markers in 215 Patients with Gastric Carcinoma.

Variables	Hazard ratio	95% CI*	*P* **
PKCα overexpression			
Negative	1		
Positive	0.632	0.407–0.982	0.0415
Age			
<60	1		
≧60	2.953	1.749–4.986	<0.0001
Pathologic stage			
I+II	1		
III+IV	2.310	1.052–5.073	0.0370
Nodal status			
N0	1		
N1-3	2.115	0.861–5.196	0.1025
Distant metastasis			
Absent	1		
Present	3.573	2.285–5.586	<0.0001
Local recurrence			
No	1		
Yes	3.174	1.856–5.428	<0.0001

* CI: confidence interval;

** Significance level: *P*<0.05.

## Discussion

The protein kinase C (PKC) family consists of serine-threonine kinases that act by phosphorylating specific protein substrates. PKCs are involved in regulating gene expression, proliferation, apoptosis, and migration [5]. Different PKC isoforms display cell specific patterns of distribution that reflect a variety of role of isoforms [18]. PKCα is the most important PKC isoform for the formation and progression of malignancies in various cell lines [11], and abnormal PKCα levels are found in many transformed cell lines [14]. PKCα acts as a tumor promoter in some tumors, but it functions as a tumor suppressor in others [13]. PKCα expression and its role in tumorigenesis and tumor progression have been documented in human cancers. PKCα overexpression has been reported in prostate carcinoma, endometrial carcinoma, high-grade bladder urothelial carcinoma, and hepatocellular carcinoma. The up- or downregulation of PKCα has been described in hematological malignancies [8], and PKCα downregulation has been observed in basal cell carcinoma and colon carcinoma [8], [19]–[21]. One study reported the activation of PKCα in breast cancer [22], whereas other studies have demonstrated the downregulation of PKCα protein in breast cancer [8], [13], [17]. PKCα inhibits cell growth in normal intestinal epithelial cells and pancreatic carcinoma [19]. Thus the expression patterns of PKC isoforms differ across different tissues and even within the same tissue [23]. To date, the role of PKCα expression in human cancers is not well understood, but seems to depend on tumor type.

PKCα has been hypothesized to play an important role in the carcinogenesis and metastasis of gastrointestinal cancers. PKCα protein is the most abundant isoform in gastric epithelial cells [10], although the role of PKCα in gastrointestinal tumors is not clear. With regard to intestinal cancer, one study has postulated that PKCα acts as a tumor suppressor [9], but another study has indicated that PKCα may act as both a tumor promoter and tumor suppressor [24]. In colon carcinoma, PKCα overexpression has been correlated with the migratory activity of tumor cells [23]. The first report to document the critical role of PKCα in maintaining the transformed phenotype of gastric cancer cells was published in 2004 [10]. Another study showed that PKCα promotes apoptosis of MGC80-3 gastric cancer cells [25]. Recently, we postulated that PKCα mRNA expression is upregulated, and is associated with distant metastasis in gastric carcinoma [15].

Several immunohistochemical studies have demonstrated that PKCα is overexpressed in high-grade bladder, prostate, and endometrial cancers, whereas breast, colon, and basal cell cancers display downregulation of PKCα expression [21]. Although an association between PKCα expression and gastric carcinoma has been documented, neither the clinicopathological correlations nor the prognostic significance of PKCα protein overexpression in gastric carcinoma had been studied. In this study, we tested the PKCα mRNA expression in gastric carcinoma at first via quantitative real-time PCR using ten pairs of tumor and non-tumor gastric tissues. Our data demonstrated PKCα mRNA expression was upregulated in gastric carcinoma. Then we applied immunohistochemical method to evaluate the expression of PKCα protein in gastric carcinomas. Our data indicated PKCα protein overexpression in 41% cases of gastric carcinoma. Furthermore, PKCα protein overexpression was correlated to clinicopathological parameters. We found PKCα protein overexpression to be statistically correlated with histologic type. Intestinal type tumors more frequently expressed PKCα protein than did diffuse type tumors. According to general concept of gastric carcinogenesis, intestinal type and diffuse type carcinomas appear to evolve through different pathways, involving different oncogenes and tumor suppressor genes [26]. Gene expression profiling studies have shown that diffuse type carcinoma exhibits an altered expression of genes related to cell-matrix interaction and extracellular-matrix components, whereas intestinal type carcinoma exhibits enhanced cell growth [27]. Thus we conduct that PKCα protein plays a role in gastric carcinogenesis, especially intestinal type carcinoma.

We also found PKCα protein overexpression to be statistically correlated with tumor differentiation. Well to moderately-differentiated tumors more frequently expressed PKCα protein than did poorly-differentiated ones. The association between PKCα activity and tumor differentiation and/or histological grading has been reported for various malignancies. In superficial bladder cancer, abnormally activated PKCα may play a role in tumor differentiation, and elevated PKCα activation correlates with higher histological grade [28]. PKCα is highly expressed in poorly-differentiated hepatocellular carcinoma cell lines [11]. In melanomas, PKCα activation is typically associated with decreased differentiation [12]. PKCα expression is elevated in high-grade endometrial tumors [19]. In breast cancer, expression of PKCα correlates with high histological grade and proliferation rate [17]. By contrast, one study reported that ovarian carcinoma exhibited decreasing in PKCα expression with increasing histological grade [29]. We found PKCα protein overexpression to be associated with histological grade and tumor differentiation in gastric carcinoma. In addition, we found that PKCα-positive high-grade dysplastic glands, precursor lesions of intestinal type carcinoma, were frequently observed in intestinal type carcinomas with PKCα protein overexpression. The PKCα protein is thus thought to be involved in the early stage of gastric carcinogenesis.

PKCα has been thought to play an important role in tumor progression. It has been implicated in several cancer-related processes, such as invasion and metastasis [10]. The role of PKCα in regulating tumor growth and development is clearly complex and highly tissue-dependent. In some cases PKCα acts as a tumor promoter, and in others it functions as a tumor suppressor [13]. In current immunohistochemical study, expression of PKCα protein was negatively statistically correlated to depth of invasion, angiolymphatic invasion, pathologic stage, and distant metastasis. We thus conduct that PKCα protein acts as a tumor suppressor, and downregulates gastric carcinoma progression.

PKCα has been reported to be a prognostic marker in human cancers. In Kong’s study, high level PKCα predicted a shortened recurrence-free survival in patients with superficial bladder carcinomas [28]. Haughian et al demonstrated that PKCα level may be a prognostic indicator of aggressive endometrial cancers [19]. Patients with higher PKCα mRNA expression in hepatocellular carcinomas have a significantly decreased survival rate [21]. For patients with breast cancer, the prognostic significance of PKCα is controversial. Lønne et al reported that patients with PKCα-positive breast carcinoma had a poorer survival rate [17], but Kerfoot et al found that PKCα was downregulated in advanced breast carcinoma [16]. Although no statistical significance via Kaplan-Meier method, our study showed a tendency for patients with PKCα protein overexpression to have a longer overall survival and disease free survival than those without overexpression. Furthermore, we found that PKCα protein overexpression was a significant independent prognostic factor for gastric carcinoma in multivariate analysis. Patients with PKCα protein overexpression had a statistically significant longer survival period.

In our previous study, we demonstrated that PKCα mRNA expression was upregulated and associated with distant metastasis in gastric carcinoma, and that PKCα mRNA overexpression predicted poor outcome [15]. Considering the results of that study together with those of the current one, we concluded that in patients with advanced gastric carcinomas, PKCα mRNA plays a promoting role in decreased survival, whereas PKCα protein has an opposing effect to suppress cancer progression and decrease cancer mortality. Several hypotheses might account for this finding. First, PKCα protein is subjected to complete proteolysis during or preceding late-stage gastric cancers. A previous study has documented that the activation and degradation of PKC isoforms were controlled spatially and temporally [30]. Second, post-transcriptional processing and RNA splicing might be responsible for the opposite effects of mRNA versus the protein of PKCα. In addition, the catalytically-competent PKCα molecules in cells are tightly regulated by phosphorylation, cofactor binding, and intracellular localization. PKCα biological activity is modulated by and functionally interacts with a number of proto-oncogenes. The PKCα molecules must be processed by a series of phosphorylation to attain catalytic competence. Undergoing translocation to the plasma membrane, PKCα is activated and consequently carries out substrate phosphorylation. After this, phosphorylated PKCα resides in the cytoplasm of the cell and requires additional regulatory mechanisms to become fully catalytically active [7], [16]. Therefore, PKCα protein expression might not fully represent kinase activity. In addition, immunoreactivity might not fully reflect true protein expression [31]. Further studies using kinase activity assay, immunogold labeling to identify subcellular localization and translocation of PKCα protein, and fluorescence in situ hybridization to detect PKCα mRNA in different pathological stages of gastric carcinoma are needed to clarify the discrepant roles of protein and mRNA of PKCα.

In conclusion, we demonstrated that PKCα protein is upregulated in gastric carcinoma. PKCα protein expression was statistically correlated with age, histologic type, tumor differentiation, depth of invasion, angiolymphatic invasion, pathologic stage, and distant metastasis. The PKCα protein overexpression was a significant independent prognostic factor for patients with gastric carcinoma in multivariate Cox regression analysis.
